# Membrane Order Effect on the Photoresponse of an Organic Transducer

**DOI:** 10.3390/membranes13050538

**Published:** 2023-05-22

**Authors:** Vito Vurro, Matteo Moschetta, Gaia Bondelli, Samim Sardar, Arianna Magni, Valentina Sesti, Giuseppe Maria Paternò, Chiara Bertarelli, Cosimo D’Andrea, Guglielmo Lanzani

**Affiliations:** 1 Center for Nano Science and Technology @PoliMi, Istituto Italiano di Tecnologia, 20134 Milan, Italy; vito.vurro@iit.it (V.V.); matteo.moschetta@iit.it (M.M.); samim.sardar@iit.it (S.S.); arianna.magni@polimi.it (A.M.); valentina.sesti@polimi.it (V.S.); giuseppemaria.paterno@polimi.it (G.M.P.); chiara.bertarelli@polimi.it (C.B.); cosimo.dandrea@polimi.it (C.D.); 2 Department of Physics, Politecnico di Milano, 20133 Milan, Italy; 3 Department of Chemistry, Materials and Chemical Engineering “Giulio Natta”, Politecnico di Milano, 20133 Milan, Italy

**Keywords:** cell passage, membrane order, thermal stimulation, optostimulation, organic materials

## Abstract

Non-genetic photostimulation, which allows for control over cellular activity via the use of cell-targeting phototransducers, is widely used nowadays to study and modulate/restore biological functions. This approach relies on non-covalent interactions between the phototransducer and the cell membrane, thus implying that cell conditions and membrane status can dictate the effectiveness of the method. For instance, although immortalized cell lines are traditionally used in photostimulation experiments, it has been demonstrated that the number of passages they undergo is correlated to the worsening of cell conditions. In principle, this could impact cell responsivity against exogenous stressors, including photostimulation. However, these aspects have usually been neglected in previous experiments. In this work, we investigated whether cell passages could affect membrane properties (such as polarity and fluidity). We applied optical spectroscopy and electrophysiological measurements in two different biological models: (i) an epithelial immortalized cell line (HEK-293T cells) and (ii) liposomes. Different numbers of cell passages were compared to a different morphology in the liposome membrane. We demonstrated that cell membranes show a significant decrease in ordered domains upon increasing the passage number. Furthermore, we observed that cell responsivity against external stressors is markedly different between aged and non-aged cells. Firstly, we noted that the thermal-disordering effect that is usually observed in membranes is more evident in aged cells than in non-aged ones. We then set up a photostimulation experiment by using a membrane-targeted azobenzene as a phototransducer (Ziapin2). As an example of a functional consequence of such a condition, we showed that the rate of isomerization of an intramembrane molecular transducer is significantly impaired in aged cells. The reduction in the photoisomerization rate translates in cells with a sustained reduction of the Ziapin2-related hyperpolarization of the membrane potential and an overall increase in the molecule fluorescence. Overall, our results suggest that membrane stimulation strongly depends on membrane order, highlighting the importance of cell passage during the characterization of the stimulation tools. This study can shine light on the correlation between aging and the development of diseases driven by membrane degradation as well as on the different cell responsivities against external stressors, such as temperature and photostimulation.

## 1. Introduction

The target of exogenous biological stimulation is to perturb the electrical state of cells with the broad aim of understanding and possibly improving/restoring biological functions. Among the various approaches, photostimulation has recently attracted increasing interest, owing to the possibility of perturbing cellular activity remotely with high spatiotemporal precision [1,2,3,4,5,6,7]. Apart from optogenetics, an alternative photostimulation paradigm relies on exogenous light-responsive materials, usually in molecular or nanostructured forms [8,9,10]. In particular, we here refer to a family of amphiphilic photochromic azobenzenes, recently demonstrated to be molecular transducers [11,12,13,14,15,16,17,18]. Optical modulation of cell parameters stems from the excitation of the azobenzene that in turn releases energy to the membrane, possibly changing the membrane thickness [19]. Being a non-genetic photostimulation method, it poses less invasiveness and fewer ethical issues than optogenetics while maintaining a comparable spatiotemporal excitation precision, although it can be elusive as it depends on relatively weak non-covalent interactions and, importantly, on membrane thermodynamics parameters. The latter parameters are fluidity, order (phase), and polarity. These parameters in living cells are influenced by both physical parameters (e.g., temperature) and the structural component of the plasma membrane, such as cholesterol concentration and phospholipid composition. They are also mutually interconnected, as (i) fluidity is usually associated with lipid packing and structural organization and it can be evaluated by the overall ratio between liquid-ordered (Lo) and liquid-disordered (Ld) domains, and (ii) the membrane phase influences the polarity of the bilayer itself. Ordered and well-packed domains (Lo) are characterized by a low polarity, as a limited amount of water is present within the bilayer. On the contrary, disordered domains (Ld) present a higher polarity, induced by the presence of water molecules intercalating inside the lipid bilayer [20,21]. It is thus crucial to carefully investigate the nature and the rationale underpinning the phototransducer–membrane cross-talk, in terms of interaction and excitation [15,22]. In this regard, an aspect that has usually been neglected is represented by cell physiological conditions as a function of cell passages during growth. The cell passage number refers to the record of the number of times the cell culture is subcultured into multiple “daughter” cell cultures typically used for immortalized or tumoral cell lines. Although immortalized cell lines are widely used in biological experiments, it is known that the cell conditions continue to worsen as the cell passage number increases [23,24,25,26,27]. Hence, two fundamental questions arise. The first is related to the possible link between the number of passages and the membrane thermodynamics parameters, while the second is based on how such changes would impact the partitioning, the localization, and the stability of the photoactuator in the membrane. The former question is somehow general, as this relationship can have broad implications for cell aging and perhaps on the development of age-related diseases [28,29,30,31], while the latter is strictly connected to the cell response against external stimuli. The work presented herein aims at answering the abovementioned questions. By using optical spectroscopy and imaging, we observed that in immortalized cells (HEK-293T) the membrane order decreases as the number of passages increases. Interestingly, we also noted that the effectiveness of membrane potential modulation, which was driven by the light excitation of an intramembrane azobenzene, was substantially decreased in aged cells. Such an effect was also reconstructed in model membranes, in which the membrane order was modulated through the increase/decrease in the cholesterol content. This study not only shows the relationship between cell aging and membrane status, but also provides a general rationale for the design and interpretation of experiments involving the use of exogenous cell-targeting materials.

## 2. Materials and Methods

**Ziapin2**

**Scheme 1:**
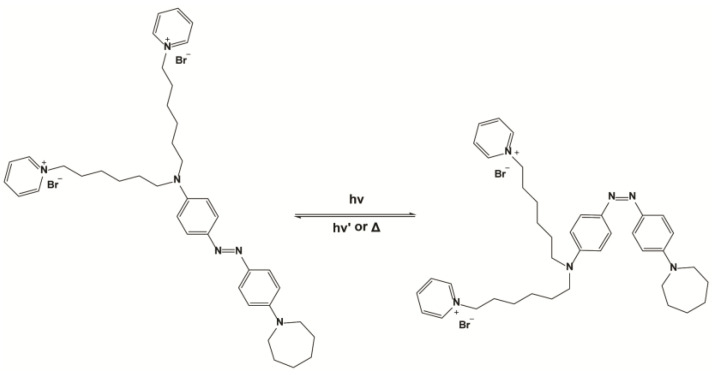
Chemical structure of Ziapin2 in both trans and cis conformation.

The synthesis of Ziapin2 consists of the reduction of the nitro group of the Disperse Orange 3 dye in amine, which is then alkylated with α,ω-dibromohexane and finally substituted with pyridine to obtain the terminated pyridinium bromide (Scheme 1). The synthesis is reported in full in DiFrancesco et al. [12]. Under dark conditions, Ziapin2 preferentially dwells inside the membrane persisting in the trans configuration. After the partitioning, Ziapin2 forms dimers leading to a thinning of the membrane and the consequent increase in the cell capacitance. Illumination induces a trans-to-cis isomerization that leads to the breaking of Ziapin2 dimers and plasma membrane relaxation. This translates into a rapid hyperpolarization followed by a slight depolarization after the end of the stimulus. This phenomenon has been reported in a variety of different types of cells. In excitable cells, such as neurons and cardiomyocytes, the membrane potential variation is sufficient to trigger action potentials [12,18].

**Cell culture maintenance**

In vitro electrophysiological experiments were performed using the immortalized cell line HEK-293T (Human Embryonic Kidney), purchased from ATCC. HEK-293T cells were cultured in T-25 cell culture flasks containing Dulbecco’s Modified Eagle Medium high glucose (DMEM-HG) culture medium, supplemented with 10% heat-inactivated FBS and 1% GlutaMAX (200 mM, Invitrogen). Culture flasks were maintained in a humidified incubator at 37 °C with 5% CO_2_. When at confluence, cells were enzymatically detached from the flasks with a 1× trypsin–EDTA solution, plated on sterilized substrates, and left to grow for 48 h before the recordings. Prior to cell plating, a layer of fibronectin (2 μg mL^−1^ in PBS solution) was deposited on the sample surface and incubated for 1 h at 37 °C to promote cellular adhesion. Cells at different passages were obtained from the same original batch purchased from ATCC. During maintenance, cells were detached and replated in the flask every 2/3 days without intermediate freezes.

**Laurdan staining process**

Laurdan (6-lauryl-2-dimethylamino-naptha-lene) was purchased from Sigma-Aldrich, Gillingham, UK. For confocal imaging and spectral measurements, a 5 mM stock solution of Laurdan in dimethyl sulfoxide (DMSO, Sigma-Aldrich, Gillingham, UK) was prepared. Laurdan staining of live HEK-293T cells was performed by removing the medium from the cell culture dish, prepared as described in the cell culture section above, and replacing it with 1 mL of fresh, serum-free DMEM, as the presence of serum increases the background fluorescence and sequesters the dye during incubation. Laurdan was then added at a final concentration of 10 μM, known to efficiently stain cells without affecting their viability. Thirty-one cells were incubated at 37 °C under a humidified 5% CO_2_ atmosphere for 30 min in the dark.

**Confocal Image Acquisition**

Laurdan-stained cell samples were mounted on an inverted confocal laser-scanning microscope (Nikon Eclipse Ti2, Nikon Instruments, Tokyo, Japan), and acquisitions were performed using an Olympus 60× oil objective. Cells were excited with a 403 nm diode laser, and the fluorescence was collected in two detection channels: 425–475 nm and 500–550 nm. Confocal images were analyzed with Fiji (ImageJ), and the generalized polarization (GP) fluidity map was realized through MATLAB.

**Fluorescence Spectral Acquisition**

Laurdan-stained cell samples were placed on the stage of a multispectral fluorescence microscope, and the Laurdan fluorescence was obtained under excitation at 370 nm. The light source was provided by a Ti:Sapphire oscillator (Chameleon Ultra II, Coherent, Santa Clara, CA, USA) producing pulses of 140 fs with a repetition rate of 80 MHz. Second harmonic generation was obtained using a barium borate crystal; two absorbing BG40 filters removed residuals of the fundamental. The excitation beam was reflected off a suitably chosen dichroic mirror (LP435) before being coupled to the objective and focused onto the sample, obtaining an excitation spot diameter of 4–5 μm and an average power of 25 μW. To achieve high excitation efficiency, a 63× water immersion objective (Leica HCX APO L 63×) with a high numerical aperture (NA 0.9) was required. Sample emission was collected by the same objective and transmitted through the dichroic mirror, and, furthermore, an LP395 absorbing filter was used to remove the residual pump scatter. The microscope’s field of view was selected by a flip mirror and a CMOS camera (ORCA-Flash 2.8, Hamamatsu Photonics, Hamamatsu City, Japan), allowing for accurate positioning of the sample relative to the excitation beam via a sample XYZ differential micrometer translation stage. The emission signal was focused on the entrance slit of a spectrograph (Acton SP2300i, Princeton Instrument, Trenton, NJ, USA). The dispersed image was then focused on the entrance slit of a streak camera (Hamamatsu C5680), equipped with the Synchroscan sweep module. Each measurement lasted no more than 2 min, in order to reduce cellular stress during excitation. Because of the high degree of intrinsic cell membrane heterogeneity, the recorded spectra slightly differed from cell to cell and from different spots of the same cell. For this reason, the cell area of interest was fixed when performing consecutive measurements at increasing temperatures, in order to avoid influencing the Laurdan response by changing the cell spot under investigation. The shape and general aspect of each sample cell were checked after each measurement through the wide-field CMOS camera, in order to ensure that the cell did not undergo any change in shape or position. For each sample, at least three measurements were performed, and the samples were changed after about 40 min from the end of Laurdan incubation, in order to avoid excessive internalization.

**Electrophysiology**

Standard patch clamp recordings were performed with an Axopatch 200B (Axon Instruments, San Jose, CA, USA) coupled with a Nikon Eclipse Ti inverted microscope. HEK-293T cells were measured in the whole-cell configuration with freshly pulled glass pipettes (4–7 MΩ), filled with the following intracellular solution (mM): 12 KCl, 125 K-Gluconate, 1 MgCl_2_, 0.1 CaCl_2_, 10 EGTA, 10 HEPES, and 10 ATP-Na_2_. The extracellular solution contained (mM) 135 NaCl, 5.4 KCl, 5 HEPES, 10 Glucose, 1.8 CaCl_2_, and 1 MgCl_2_. The acquisition was performed with pClamp-10 software (Axon Instruments, San Jose, CA, USA). Membrane currents were low pass filtered at 2 kHz and digitized with a sampling rate of 10 kHz (Digidata 1440A, Molecular Devices, San Jose, CA, USA). Current-clamp recordings were acquired and analyzed only in cells with an access resistance lower than 8 MΩ. Both capacitance and resistance were compensated for. A cyan LED coupled to the fluorescence port of the microscope and characterized by the maximum emission wavelength at 474 nm provided the excitation light source. The illuminated spot on the sample had an area of 0.23 mm^2^ and a photoexcitation density of 27 and 53 mW/mm^2^, as measured at the output of the microscope objective. HEK-293T cells were loaded with Ziapin2 at a fixed concentration of 25 µM for 7 min before performing the electrophysiological recordings. Ziapin2 was synthesized according to the procedure reported in Vurro et al. [14] and purity was assessed by ^1^H and ^13^C-NMR (Bruker ARX400). For all the reported experiments, Ziapin2 was resuspended in MilliQ water at an initial concentration of 2 mM. Data were analyzed with GraphPad Prism 6 software (GraphPad Software, Inc., La Jolla, CA, USA). 

**Statistical analysis**

Normal distribution was assessed using D’Agostino and Pearson’s normality test. Data are expressed as means ± SD for n = 3 independent experiments. The errors on derived quantities, such as GP, were calculated by error propagation. Data expressed as box plots show the following elements: center line, median (Q2); square symbol, mean; box limits, 25th (Q1) to 75th (Q3) percentiles. The whisker length was determined by the minimum and maximum values. To compare more than two normally distributed samples, one-way ANOVA or Kruskal–Wallis followed by post hoc multiple comparison tests were used. The significance level was preset to *p* < 0.05 for all tests. Statistical analysis was carried out using Prism 6 (GraphPad Software, Inc.).

**Liposome preparation**

We started with lipid powders purchased from Avanti Polar Lipids Inc. of the following lipids: 1-palmitoyl-2-oleoyl-glycero-3-phosphocholine (POPC), egg sphingomyelin (SM), and cholesterol from ovine wool (Chol). The protocol for the preparation of liposomes is described by the lipid supplier. Briefly, we dissolved the lipids in chloroform (CHCl_3_, Sigma Aldrich) at 100 mg/mL, then transferred 25 mg of lipids to a round-bottom flask, together with 1 mL of CHCl_3_ and 1 mL of methanol (CH_3_OH). The lipid mixture was dried out by means of a rotary evaporator at around 40 °C at 150 rpm for three hours. The dry lipid mixture film was left to rest overnight at −20 °C. The mixture was then hydrated with 1 mL of buffer solution (10 mM Tris (tris(hydroxymethyl) aminomethane) and 100 mM NaCl). In order to favor hydration, the lipid suspension was subjected to seven freeze–thaw cycles by alternately placing the sample in liquid nitrogen and in a warm water bath (at around 60 °C). The lipid mixture, now containing multilamellar vesicles, was extruded through a porous polycarbonate (PC) membrane, whose pore diameter was 100 nm, several times. As the extrusion needs to be carried out at a temperature higher than the phase transition of all the components, we set the temperature to around 50 °C. The liposome suspensions we obtained after extrusion were at a concentration of 25 mg/mL. All of the spectroscopic characterizations were carried out at a concentration of 5 mg/mL, diluting the product of the extrusion with the buffer. The liposome solutions can be stored at 4 °C and are stable for around ten days.

**Transient absorption measurements**

Ultrafast transient absorption measurements were performed using a Ti:Sapphire laser with 2 mJ of output energy, a 1 kHz repetition rate, a pulse width of 100 fs, and a central wavelength of 800 nm. Samples were pumped with 490 nm light generated using a visible optical parametric amplifier (OPA). Pump pulses were focused on a 200 μm spot (diameter), keeping a power of 100 μW. The white-light probe pulse was generated with a sapphire plate. The sample was contained in a quartz cuvette, so that the optical path length was 1 mm. The solution was continuously fluxed by means of a peristaltic pump, in order to ensure that always-fresh samples were exposed to light. The transmitted probe signal was collected by an optical multichannel analyzer (OMA).

## 3. Results

### 3.1. Membrane Disordering

We assessed the membrane order status using a well-known membrane probe called Laurdan. This is an amphiphilic molecule sensitive to the polarity of the membrane environment [32]. Specifically, Laurdan is intrinsically non-fluorescent in water, while it affords radiative emissions when it intercalates into a lipid bilayer. The mechanism of action relies on the fact that the Laurdan is characterized by a covalent and apolar excited state and by a charge transfer state, with the latter being strongly stabilized by the presence of water molecules in the phospholipid bilayer producing a shift in the emitted photons toward a lower energy. This property defines the Laurdan emission spectrum that peaks at around 460 nm in non-polar solvents, while another peak appears at longer wavelengths (~510 nm) as the polarity increases. Such spectral features can be taken as a biophysical signature of the membrane state [33,34,35]. Indeed, a more ordered environment corresponds to a lower water content, i.e., it is less polar than disordered membranes, which allows for a higher level of water penetration [36]. This solvatochromic property makes Laurdan suitable for quantitative membrane order assessment by evaluating the general polarization (GP) parameter, defined as:

$$GP=\frac{I_{B}-I_{G}}{I_{B}+I_{G}}$$

where I_B_ and I_G_ are the intensities at 460 nm (Lo domain) and 510 nm (Ld domain), respectively, obtained by the deconvolution of the emission spectrum. Furthermore, GP has been demonstrated to be independent from the nature of the phospholipid polar head group giving a precise estimation of the overall plasma membrane order [37]. GP values always lie between −1 and 1. The higher the GP, the more ordered the membrane.

**Figure 1:**
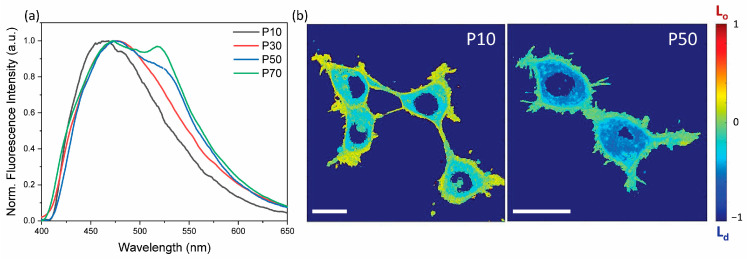
Laurdan emission cell passage dependency. (**a**) Evolution of Laurdan emission spectra as a function of the cell passage number. (**b**) Confocal images of a whole cell’s membrane order map as a function of cell passage (scale bar = 10 µm).

**Figure 2:**
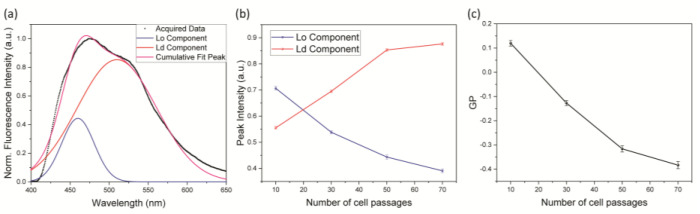
GP value extraction and cell passage number dependency. (**a**) Laurdan spectrum deconvolution. (**b**) Evolution of the Laurdan emission spectrum components as a function of cell passage number. (**c**) Evolution of the GP as a function of cell passage number. Data (represented as mean ± SD) were obtained by 3 independent preparations.

By using this tool, we evaluated the membrane order as a function of the cell passage number (P). Figure 1 shows that an increase in P corresponds to an enhancement of the spectral component at 510 nm, implying that the cell membrane becomes more polar and disordered with cell aging. Quantitative information was extracted by fitting the emission spectra at different P values with two Gaussians, centered at 460 nm and 510 nm (Figure 2a). The intensity of the two peaks was plotted as a function of P, clearly showing an inversion of the dominant membrane phase (Figure 2b), with the ratio between the amplitude of the two components growing linearly with P (linear fit R^2^ = 0.987) (Figure S1). Finally, starting from this value, we calculated the GP by exploiting the equation previously described (Supporting Table S1). Again, we confirmed a decrease in the membrane order even though the trend seemed to be linear only during the initial cell aging process (Figure 2c). These observations clearly indicate that the membrane phase becomes less ordered with cell aging.

### 3.2. Thermal Effect

**Figure 3:**
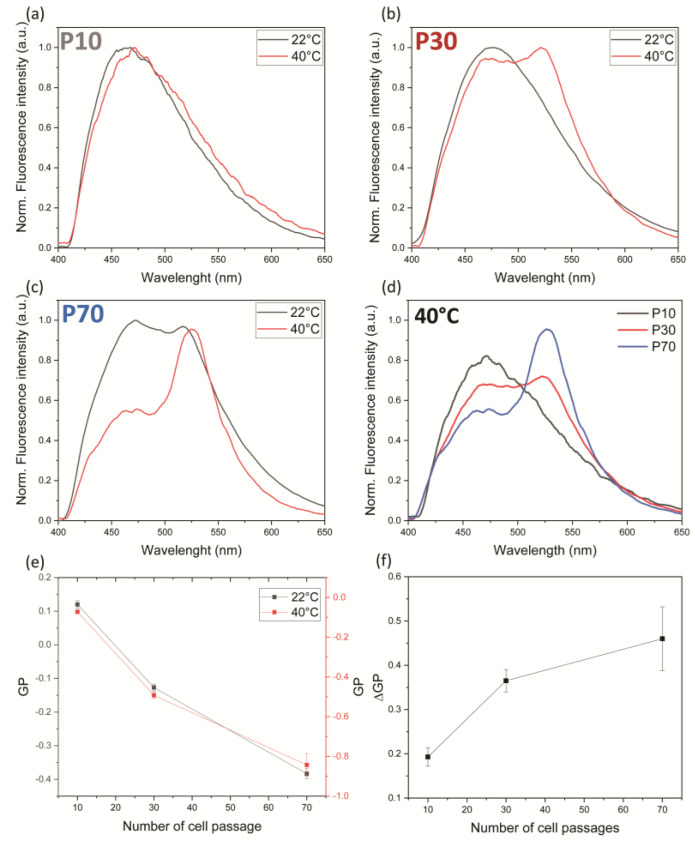
Effect of temperature variation on Laurdan emission spectra and GP as a function of cell passage number. (**a**–**c**) Laurdan emission spectra measured at different temperatures and different cell passage numbers. The cell passage is reported in each panel (a-P10, b-P30, and c-P70) and the temperature is reported in the following color code: black curves for 22 °C and red curves for 40 °C. (**d**) Comparison between Laurdan emission spectra at 40 °C as a function of the cell passage number. (**e**) Evolution of the GP as a function of the cell passage number at 22 °C (black line) and 40 °C (red line). (**f**) GP variation between 40 °C and 22 °C as a function of the cell passage number. Data (represented as mean ± SD) were obtained by 3 independent preparations.

After having shown that at each passage the cell membrane structure changes, we tested how this affects the membrane response to thermal stimulation. We employed a Peltier plate, whose voltage was adjusted via a source meter (Agilent, B2912A), to induce in HEK-293T cells a temperature variation of 18 °C, starting from an initial value of 22 °C, in samples at different P values. Figure 3 shows that the temperature increase affects the cell membrane at any passage number, leading to more fluid lipid bilayers. However, this change is much more dramatic in cells at larger P values. Indeed, in cells at P = 70 at 40 °C, the Ld component (510 nm) becomes the dominant spectral feature. Accordingly, in all cases heating produces a shift in the GP (reported in Supporting Table S2) towards more negative values, confirming the expected disordering effect upon the increase in temperature. A plot of the GP variation ($∆GP=\left|{GP}_{40{}^{\circ}C}-{GP}_{22{}^{\circ}C}\right|$) [35] displays the increased response to heating at higher P values (Figure 3f). Membranes that are more ordered at room temperature are less prone to change if subjected to thermal stress. This effect could be ascribed to reduced segregation of ordered and disordered domains, which agrees with the measured variation in the GP parameter [38]. This behavior has already been reported in the literature confirming that cell aging is a good platform for testing the photostimulation efficiency in cells with different membrane orders [39].

### 3.3. Relationship between Photoactuation and Cell Passage

**Figure 4:**
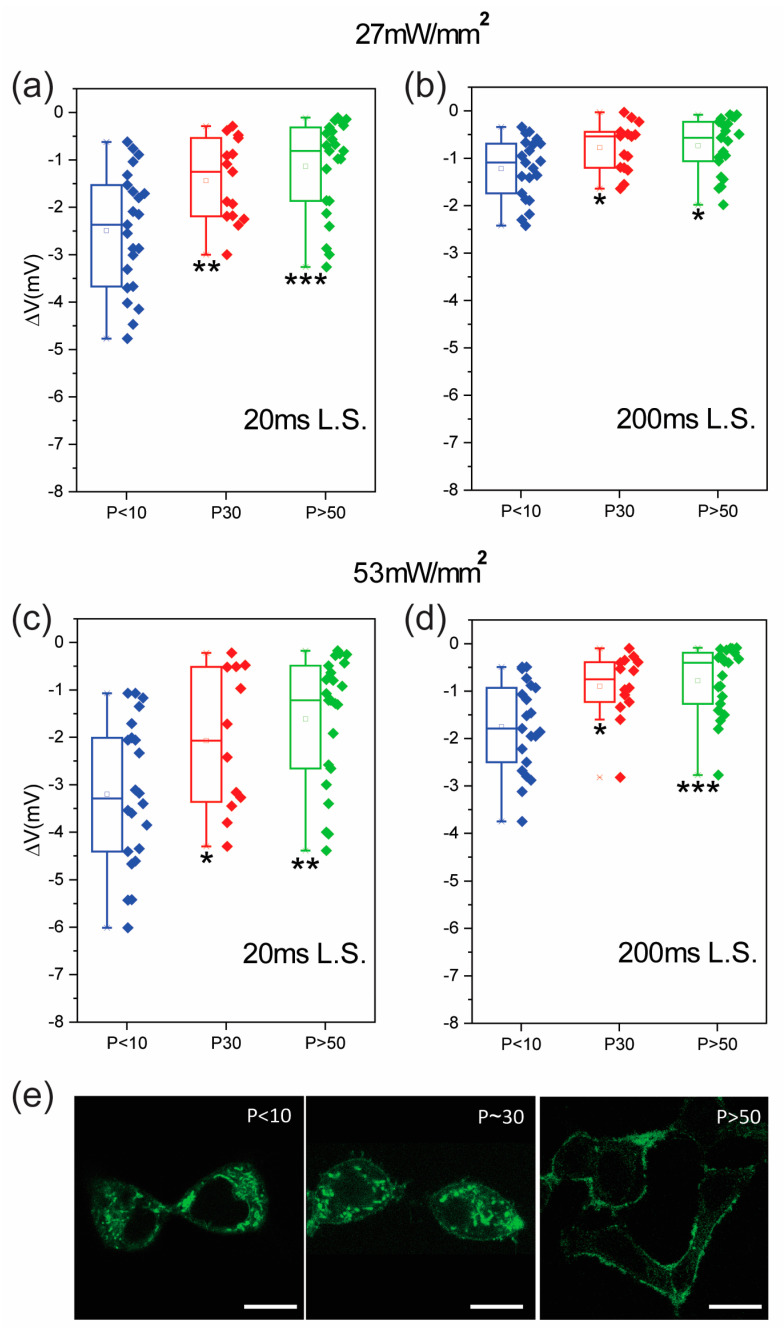
Ziapin2-based photostimulation dependency on the cell passage number. Plots representing the amplitude of the hyperpolarizing phase at increasing cell passages. Light stimulation persists for both 20 ms (**a**,**c**) and 200 ms (**b**,**d**). Squares represent separate data points. (**e**) Confocal images of Ziapin2-treated HEK-293T cells at different cell passages (scale bar = 10 µm). Data are shown as box plots. Holm–Sidak’s multiple comparison test after a one-way ANOVA test or Dunn’s multiple comparison test after a Kruskal–Wallis test. * *p* < 0.05; ** *p* < 0.01; *** *p* < 0.001. 53 mW/mm^2^: n = 22, 12, and 23 (20 ms); n = 21, 14, and 21 (200 ms); 27 mW/mm^2^: n = 23, 15, and 23 (20 ms); n = 21, 15, and 21 (200 ms) for *p* < 10, P = 30, and *p* > 50, respectively.

The observed change in the membrane structure with P and the variation in the membrane response to heating invited us to explore how P affects the cell reaction to another exogenous stimulus, namely photostimulation via a recently introduced intramembrane azobenzene (Ziapin2). This amphiphilic molecule is able to partition stably in apolar lipid membranes and isomerizes when irradiated [12,13,14,15]. On the contrary, in a polar environment, such as water, Ziapin2 is packed into aggregates that hamper isomerization. During partitioning, Ziapin2 dimerization induces a thinning of the plasma membrane with a consequent increase in the membrane capacitance. Isomerization in the membrane leads to a rapid hyperpolarization of the membrane potential that is associated with a change in the plasma membrane capacitance possibly triggered by a relaxation in the membrane thickness [12]. This phenomenon should then be influenced by the membrane structure, and in turn by P. In order to test this hypothesis, we loaded HEK-293T cells at different passages (P < 10, 30, and >50) with Ziapin2 and acquired the cell response by the whole-cell patch-clamp technique (Figure 4 and Figure S3). We noted that although the electrophysiological effect was observed at all cell passages, the amplitude of the hyperpolarization phase was significantly reduced in cells at higher P values. In particular, HEK-293T cells at P < 10 exhibited a hyperpolarization of −3.2 ± 0.33 mV when stimulated for 20 ms at the maximum power density (53 mW/mm^2^). On the other hand, cells at higher passages in the same experimental conditions showed a hyperpolarization amplitude of −2.07 ± 0.43 mV and ~−1.61 ± 0.28 mV for P = 30 and P > 50, respectively. The same phenomenon occurred under a long-lasting stimulus (200 ms) and a lower power density (27 mW/mm^2^). These results could stem from a change in the photoisomerization dynamics or in the membrane response to isomerization. In a previous study, we observed a significant reduction in the Ziapin2-induced photomodulation in neuronal cells treated with β-cyclodextrin [12]. This compound reduces the amount of cholesterol inside the plasma membrane favoring the disruption of the lipid rafts and inducing a subsequent decrease in the membrane order. These observations are consistent with the results in Figure 4 and corroborate the hypothesis about the role of the membrane environment. Furthermore, we also evaluated the dispersion in the amplitude of the membrane potential modulation as a function of the cell passage (Supporting Figure S2), observing an inverse proportionality between the two quantities. We tentatively assigned this to the fact that a more disordered membrane is globally more homogeneous.

A final proof of the relation between membrane order and photostimulation efficiency was inferred from the fluorescence yield of Ziapin2. If our hypothesis about the photoisomerization–membrane order relation is correct, we would expect a decrease in the Ziapin2 emission when internalized in more ordered membranes. Indeed, isomerization and fluorescence are competitive processes and the branching ratio between the two is strongly influenced by the local environment experienced by the molecule. In order to test this hypothesis, we acquired confocal images of HEK-293T cells at different passages loaded with Ziapin2 (Figure 4e). We observed that in cells at lower passages (P < 10), the Ziapin2 emission was preferentially located in the cytoplasm; on the contrary, at higher cell passages (P > 50), the molecule photoluminescence specifically localizes in the plasma membrane. Considering that the cytosol can be associated with water, we hypothesize that a higher Ld phase in the membrane (meaning a higher cell passage) favors photoluminescence from the membrane over the competitive isomerization process, which is progressively hampered. Indeed, the photoisomerization of Ziapin2 that occurs when the molecule is internalized in disordered lipid bilayers competes with radiative decays. 

### 3.4. Photoactuation in Liposomes

**Figure 5:**
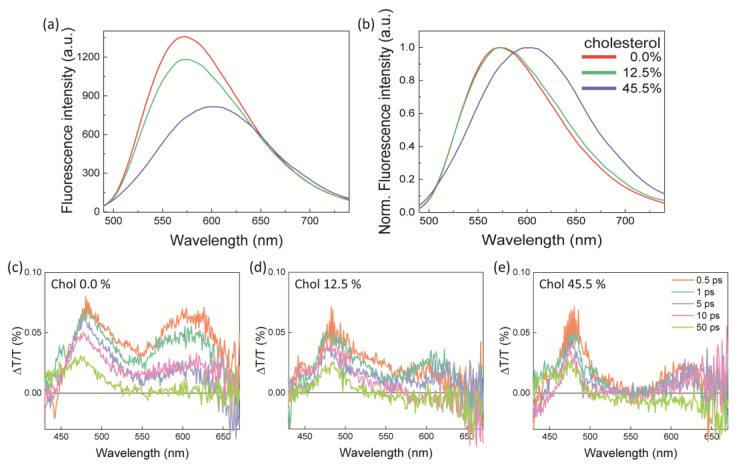
Ziapin2 in liposomes. (**a**,**b**) Fluorescence spectra of Ziapin2 in liposome suspensions as a function of the cholesterol content. (**c**–**e**) Differential transient transmittance spectra of Ziapin2 in liposome suspensions at different cholesterol contents ((**c**) 0%, (**d**) 12.5%, and (**e**) 45.5% of cholesterol) at different time delays.

Finally, we employed model membranes to study the mechanism behind the different actuation capabilities of Ziapin2 in the different membrane phases, as model systems allow for a more straightforward physical characterization than living cells. We achieved this by fabricating artificial lipid bilayers of increasing cholesterol content, since the rigid sterol causes the lipid acyl chains to become closely packed and the bilayer to be thickened [40]. Overall, this leads to an ordering effect on the Ld phase. In particular, we prepared liposomes by mixing three different lipids: a phospholipid (1-palmitoyl-2-oleoyl-glycero-3-phosphocholine, POPC), sphingomyelin (SM), and cholesterol (Chol), keeping the ratio between SM and Chol equal to 1 in all the samples, and investigated variations due to phase changes from the Ld phase to the Lo phase. We examined three different liposome compositions: (i) pure POPC liposomes (chol 0%) representing the Ld phase, (ii) POPC:SM:Chol 6:1:1 mol:mol (chol 12.5%) mimicking the coexistence of the Ld and Lo phases, and (iii) POPC:SM:Chol 1:5:5 mol:mol (chol 45.5%) reproducing the pure Lo phase [41]. We characterized Ziapin2’s isomerization ability in these samples by means of optical spectroscopy. Starting from fluorescence (Figure 5a), we observed that the PL intensity decreases as the cholesterol (and SM) molar fraction in the liposomes grows. The reduction in the fluorescence yield in the Lo phase can be attributed to the higher isomerization rate of Ziapin2. In addition, by increasing the cholesterol concentration, the emission peak shifts from 570 nm to 620 nm (Figure 5b). This feature can also be related to the different isomerization capabilities of the molecule in the different lipid phases. Specifically, the cis isomer of Ziapin2 has a (weak) fluorescence peak at around 500 nm, while the trans isomer emits at around 600 nm. Both the fluorescence yield and the PL peak shifts can thus be attributed to Ziapin2 being phase-sensitive. To gain further insight into the isomerization dynamics in the different environments, we performed ultrafast transient absorption spectroscopy. Figure 5c–e reports the transient absorption spectra at various temporal delays of Ziapin2 in the liposomes of different compositions, whose spectral features have been already assigned in recent publications [13,16]. Briefly, the positive ∆T/T signal between 460 nm and 500 nm is related to the ground state bleaching (GSB) of the trans isomer of the molecule. The spectral region between 560 nm and 620 nm exhibits a positive signal that is related to the stimulated emission (SE). Finally, there is a negative feature peaking at around 550 nm, which is a photoinduced absorption (PIA) band, usually associated with the main absorption band of the cis isomer of Ziapin2. We noticed that, by increasing the amount of cholesterol in the liposomes, the spectral band at 510–560 nm becomes more negative, i.e., the PIA increases. Moreover, as expected, the SE signal is higher in the absence of cholesterol as the emission is enhanced in cholesterol-poor liposomes. Finally, when increasing the amount of cholesterol in the liposomes, the GSB kinetics become slower due to the formation of the long-living cis isomer of the molecule. All these features support the hypothesis of the isomerization being favored in a cholesterol-rich environment, which is a more ordered lipid membrane phase.

## 4. Discussion

It has been reported that, at higher passages, cells undergo several physiological modifications that could compromise or alter cellular functions [26,42,43,44]. However, it is not possible to determine a general law that explains this phenomenon because of the intrinsic variability in the different cell lines. In addition, the number of cell passages is often a neglected parameter in the experimental setting. Even if it is not possible to provide a unique model that describes the biological consequences of continuously passing cells, we can try to provide possible explanations for the physiological alterations that cells undergo after several passages.

In the last few decades, different works have proposed that free radicals, such as reactive oxygen species (ROS), could play a role in this complex phenomenon. It has been reported that ROS increase their concentration inside the cells at increasing passages determining lipid oxidation and protein degradation [45,46,47,48]. The rate of lipid peroxidation as well as products of oxidative modifications of proteins are known to progressively accumulate during a cell’s life even in living organisms [49,50,51,52]. In particular, it has been proposed by molecular dynamic simulation that the presence of peroxidation products decreases the order of the phospholipid bilayer by progressively altering the phospholipid structure and the lipid domains and damaging protein components [47,53]. This could lead to a general modification of phase properties of the plasma membrane, such as viscosity, polarity, and density. 

In the present work, we provided data that confirm this overall alteration in membrane physical properties upon increasing the number of passages. The cell passage number translates into a plasma membrane loss of organization with a consequent increase in the disordered phase and water content, as proved by the membrane polarity probe Laurdan. The progressive disorganization of the plasma membrane strongly affects the response of cells against external stressors. We investigated the effect of both temperature and a photoactuator on the membrane phase. In both observed cases, the increase in the number of cell passages strongly affected the stressor behavior. The thermal-disordering effect was revealed to be enhanced in cells subjected to a high number of passages, suggesting that the membrane order affects the cell response. In particular, the thermally induced GP variation is strongly dependent on the initial cell membrane order as previously reported. Moreover, another indication is provided by the standard deviation of the ΔGP. It is possible to notice that the dispersion of this value increases with the number of cell passages. This effect could be ascribed to the lesser disordering effect induced by the increase in the number of cell passages instead of the effect of temperature variation. These hypotheses are also supported by the fact that the light-dependent effect of the membrane-targeted azobenzene, Ziapin2, was impaired. Here, we saw that the disordered bilayers significantly hamper isomerization, reducing the amplitude of the light-evoked membrane hyperpolarization. On the contrary, in ordered bilayers, Ziapin2 isomerization is favored and the amplitude of the photo-induced hyperpolarization is higher. Intriguingly, the increase in the disorganization of the lipid bilayers did not monotonically grow with the number of cell passages. Focusing on the electrophysiological results, the modifications in terms of membrane organization seem to be more prominent until cells reach the 30th passage. This suggests that plasma membrane modification takes place rapidly at early stages and then seems to reach a sort of “plateau”. We can hypothesize that, at a certain point, the accumulation of defects at the level of the plasma membrane could affect the cell viability itself, determining the onset of compensatory mechanisms that maintain membrane disorganization below critical values. 

However, it is important to underline that all results were obtained in HEK293T cells, an epithelial cell line obtained by genetic modification. Despite the fact that some physiological characteristics are common in all cell types, it is not possible to generalize our findings. Indeed, cell lines derived from neuronal or muscular tissue and primary cells could exhibit different modifications under the same experimental conditions. Overall, our data show two important and perhaps overlooked effects: (i) the cell membrane is significantly altered as the number of cell passages increases, even in immortalized cell lines, and (ii) cell aging can impair the responsivity against external stressors, such as an increase in temperature or exposure to exogenous photoresponsive materials. The former effect can have a broad range of implications, as cell membrane deterioration has been linked to the development of various age-related diseases, while the latter aspect is paramount when one exposes cells to stressors, such as light, temperature, and drugs, with a view to modulating/restoring biological functions.

## Data Availability

The data presented in this study are available on request from the corresponding author.

## Supplementary Materials

The following supporting information can be downloaded at: https://www.mdpi.com/article/10.3390/membranes13050538/s1. Supporting Figure S1: Laurdan emission spectra evolution as a function of cell passage. Supporting Figure S2: The dependence of the standard deviation of the cell membrane potential modulation on the cell passage. Supporting Figure S3: Confocal images representing HEK-293T cells at different passages loaded with Ziapin2. Supporting Table S1: Supplementary tables of Laurdan GP values of HEK-293T cells at different passages. Supporting Table S2: Dependency of temperature variation.
