# Supplementary material for: Membrane Order Effect on the Photoresponse of an Organic Transducer
Source: Membranes (Basel). 2023 May 22;13(5):538. doi: 10.3390/membranes13050538 (PMC10220526; doi:10.3390/membranes13050538)
Supplement: Supplementary file 1 [file membranes-13-00538-s001.zip › membranes-2385975-supplementary.pdf]

SUPPORTING INFORMATION

Membrane Order Effect onto the Photoresponse of Organic Transducer

Vito Vurro[1]†, Matteo Moschetta[1]†, Gaia Bondelli[1,2]†, Samim Sardar[1], Arianna Magni[1,2], Valentina Sesti[1,3], Giuseppe M. Paternò[1,2], Chiara Bertarelli[1,3], Cosimo D'Andrea[1,2] and Guglielmo Lanzani[1,2]*

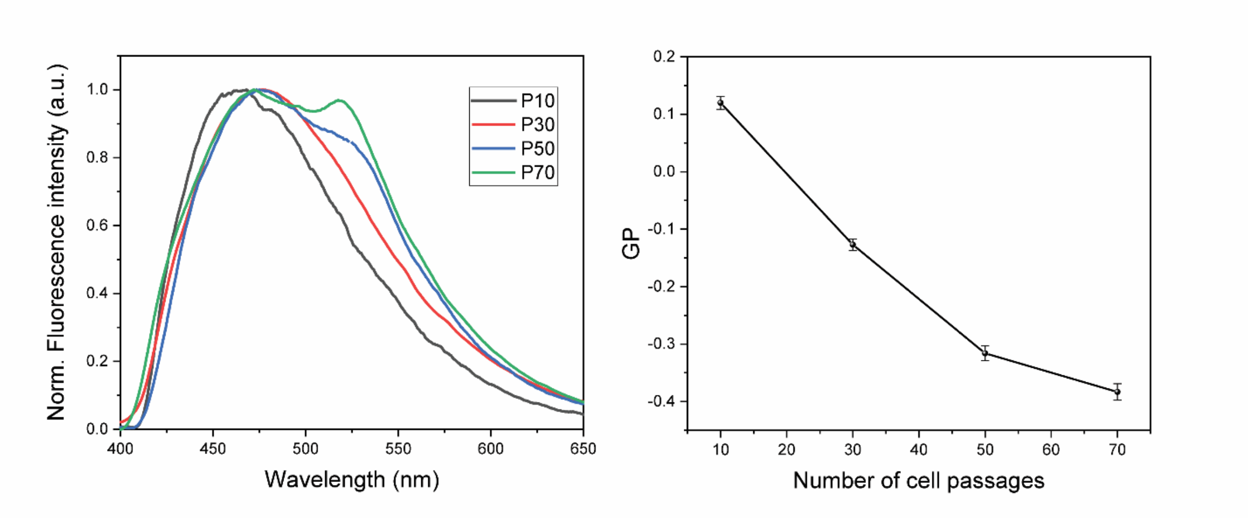

Figure S1. Laurdan Spectra evolution as a function of cell passage number. a) Laurdan emission spectra acquired in HEK-293T cells at a different passage number. b) Evolution of the extracted GP value as a function of cell passage number. Data are represented as mean ± SD.

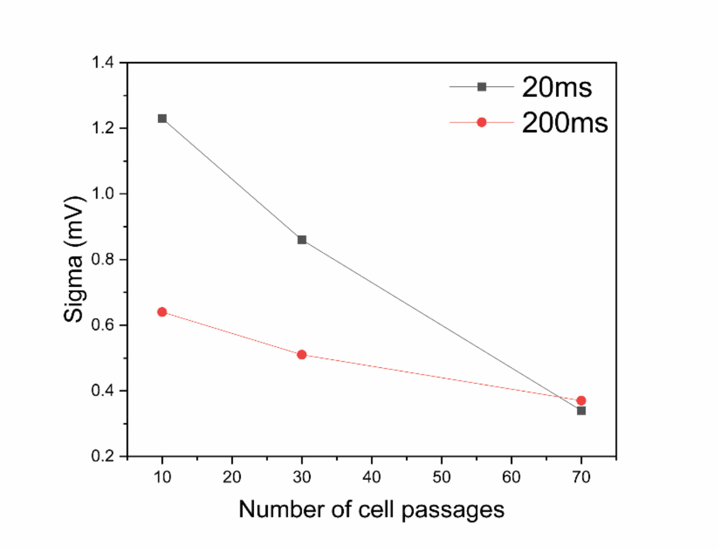

Figure S2. Standard deviation value of the cell membrane potential modulation as a function of the cell passage number. These values are reported as a results of 20ms (black line) and 200ms (red line) light pulses duration.

Table S1. Resulting values of the deconvolution process for 460nm component (I_B) and its standard deviation (σ_B) and for 510nm component (I_G) and its standard deviation (σ_G). GP and σ_{GP} value obtained by the previous parameters. All the values are extracted or calculated at different cell passage number.

	I_B	σ_B	I_G	σ_G	GP	σ_{GP}
P10	0,707	0,007	0,555	0,006	0,120	0,011
P30	0,538	0,006	0,695	0,005	-0,127	0,010
P50	0,443	0,008	0,853	0,005	-0,316	0,013
P70	0,391	0,008	0,876	0,006	-0,383	0,015

Table S2. Resulting values of the deconvolution process for 460nm component (I_B) and its and for 510nm component (I_G) obtained at 40°C. $GP_{40°}$ value was calculated by the previous parameters while $GP_{20°}$ was taken by Supporting Table 1. ΔGP and $\sigma_{\Delta G}$ was calculate as difference between GP calculated at 40°C and 22°C. All the values are extracted or calculated at different cell passage number.

	I_B	I_G	$GP_{40°}$	$GP_{22°}$	ΔGP	$\sigma_{\Delta GP}$
P10	0,465	0,538	-0,073	0,120	-0,193	0,021
P30	0,229	0,673	-0,492	-0,127	0,365	0,025
P70	0,066	0,774	-0,843	-0,383	0,460	0,072

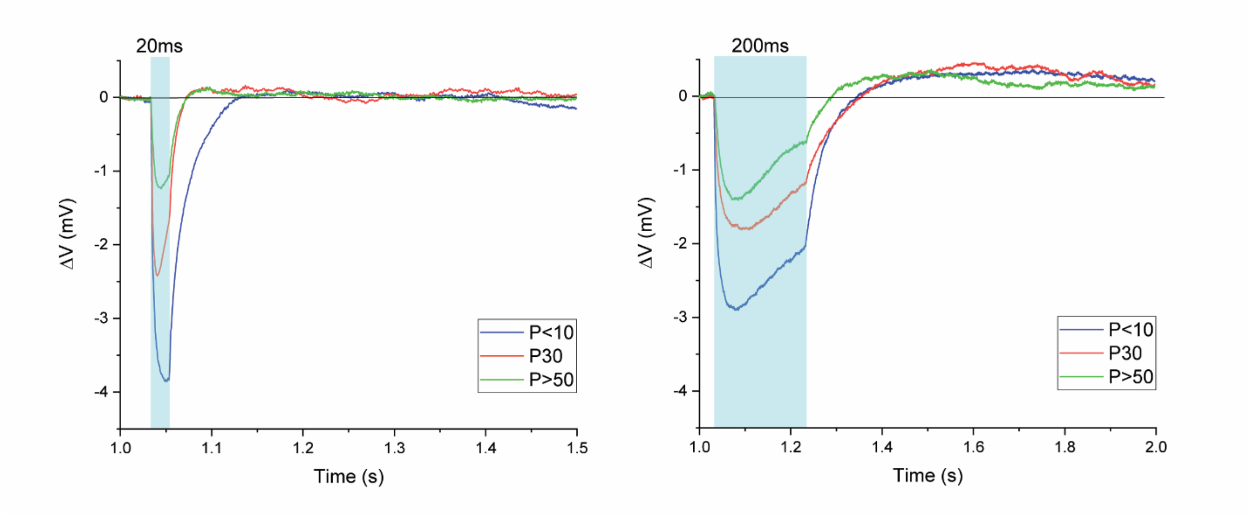

Figure S3. Representative traces of membrane potential hyperpolarization of Ziapin2-treated HEK-293T cells at increasing cell passages. Light stimulation (power density: 53mW/mm^2) persists for both 20ms (left) and 200ms (right).
